# Respiratory syncytial virus disease burden in adults aged 60 years and older in high‐income countries: A systematic literature review and meta‐analysis

**DOI:** 10.1111/irv.13031

**Published:** 2022-11-11

**Authors:** Miloje Savic, Yolanda Penders, Ting Shi, Angela Branche, Jean‐Yves Pirçon

**Affiliations:** ^1^ GSK Wavre Belgium; ^2^ Usher Institute University of Edinburgh Edinburgh UK; ^3^ Division of Infectious Diseases, Department of Medicine University of Rochester Rochester New York USA

**Keywords:** acute respiratory infection, disease burden, high‐income countries, meta‐analysis, older adults, respiratory syncytial virus

## Abstract

**Background:**

Respiratory syncytial virus (RSV)‐associated acute respiratory infection (ARI) is an underrecognized cause of illness in older adults. We conducted a systematic literature review and meta‐analysis to estimate the RSV disease burden in adults ≥60 years in high‐income countries.

**Methods:**

Data on RSV‐ARI and hospitalization attack rates and in‐hospital case fatality rates (hCFR) in adults ≥60 years from the United States, Canada, European countries, Japan, and South Korea were collected based on a systematic literature search (January 1, 2000–November 3, 2021) or via other methods (citation search, unpublished studies cited by a previous meta‐analysis, gray literature, and an RSV‐specific abstract booklet). A random effects meta‐analysis was performed on estimates from the included studies.

**Results:**

Twenty‐one studies were included in the meta‐analysis. The pooled estimates were 1.62% (95% confidence interval [CI]: 0.84–3.08) for RSV‐ARI attack rate, 0.15% (95% CI: 0.09–0.22) for hospitalization attack rate, and 7.13% (95% CI: 5.40–9.36) for hCFR. In 2019, this would translate into approximately 5.2 million cases, 470,000 hospitalizations, and 33,000 in‐hospital deaths in ≥60‐year‐old adults in high‐income countries.

**Conclusions:**

RSV disease burden in adults aged ≥60 years in high‐income countries is higher than previously estimated, highlighting the need for RSV prophylaxis in this age group.

## 1 BACKGROUND

Respiratory syncytial virus (RSV) is primarily known for its high burden of disease in infants and young children and is increasingly recognized as an important cause of serious respiratory illness in older adults and those with underlying comorbidities.^1^ RSV infections in older adults were initially described in those living in long‐term care facilities, who are often frail and have underlying diseases,^2^, ^3^ and were more recently also evaluated in healthy, community‐dwelling adults.^1^, ^4^, ^5^ While the disease is mostly mild to moderate, it can result in hospital admission, serious complications, or death.^6^

There are currently no licensed vaccines to prevent RSV‐associated disease, and there is a lack of available treatment options, which are mostly limited to supportive care.^6^ Currently, RSV diagnostic testing is not consistently done in adults upon presentation with respiratory symptoms, likely because the lack of specific antiviral therapy discourages physicians.^7^ The sensitivity of antigen‐based testing is low and/or inconsistent in adults, whereas the use of the more sensitive polymerase chain reaction (PCR)‐based testing is limited due to its relatively high costs.^7^, ^8^ Detection of RSV infections is further complicated by the lack of uniform clinical case definition for RSV and the non‐specificity of RSV symptoms.^9^ Many countries use existing influenza surveillance systems and rely on influenza case definitions to detect RSV cases, which is a suboptimal solution.^9^ These reasons contribute to an underestimation of the RSV disease burden in older adults despite the growing body of evidence indicating that it may compare to the influenza disease burden.^10^, ^11^

A recent meta‐analysis of RSV disease burden estimated that community incidence, hospitalization rate, and in‐hospital case fatality rate (hCFR) are substantial among older adults in industrialized countries (categorized based on the United Nations [UN] Children's Fund's classification in 2015).^12^ Data from developing countries were limited in this meta‐analysis. We undertook a systematic literature review and meta‐analysis to update and expand the previous review with more recent data, using a wider span in age, that is, 60 years or older, and removing non‐industrialized countries. The current analysis aimed to estimate the RSV disease burden in 2019 in high‐income countries in ≥60‐year‐old adults, in terms of RSV‐associated (1) attack rates, (2) hospitalization rates, and (3) hCFR.

## 2 METHODS

### 2.1 Systematic literature review

Studies reporting on the incidence of RSV‐associated acute respiratory infection (ARI), hospitalizations, and hCFR in ≥60‐year‐old adults, which were conducted in high‐income countries in the northern hemisphere with existing data (the United States, Canada, Europe, Japan, and South Korea), were included in this systematic literature review. Japan and South Korea were considered as high‐income countries in Asia, but no incidence data were available for South Korea. Highly relevant multicountry studies conducted in the United States, Canada, European countries, Japan, and South Korea were also included even if they comprised countries from other geographic areas.

RSV infections in the included studies had to be confirmed via PCR or fourfold or greater seroconversion (at least fourfold increase in RSV antibody titer compared with baseline) for at least the majority of cases. The included studies had to provide either incidence data or data that could be used to extract/calculate incidence data for RSV‐ARIs, hospitalizations, or hCFR. Articles were excluded if results were not presented for the age group of interest, the study population was not representative of the general population, or the publication (e.g., letters to the editor, editorials, systematic literature reviews) or research types (e.g., genetic research, molecular research) were non‐pertinent. Data on RSV‐ARI or hospitalization attack rates from retrospective studies were excluded due to the potential underreporting bias caused by the lack of systematic RSV testing in older adults. If multiple articles using the same dataset were available, only the most recent article was included in the review.

We conducted a systematic literature search in PubMed following the Preferred Reporting Items for Systematic reviews and Meta‐Analyses (PRISMA) guidelines^13^ and the Cochrane Collaboration guidelines for performing systematic reviews^14^ for articles written in English and published from January 1, 2000, to November 3, 2021. The detailed search strategy is listed in Table S1. The initial search, which identified articles published from January 1, 2000 to October 3, 2019, was supplemented by a search in Embase and CINAHL. The current systematic literature search was further supplemented with data from additional studies that authors identified by citation search, reviewing unpublished studies reported in Shi *et al*,^12^ or searching the gray literature or the abstract booklet from the RSV Vaccine for the World 2021 conference (RSVVW'21) to collect the most recent data.

Retrieved titles and abstracts were screened by one author (MS or YP). Relevant full‐text articles were selected by MS or YP, and quality control was performed by JYP. Quality control for the original search and the updates consisted of comparing the results independently extracted from approximately 25% of full‐text articles by the two authors. Differences between the results for the articles screened in duplicate were discussed to achieve alignment.

Incidence rates for RSV‐ARI cases, hospitalizations (annual hospitalization rates among all older adults), and hCFR were extracted to an excel file by MS, YP, and JYP jointly. Because most included articles estimated RSV‐ARI and hospitalization attack rates instead of incidence rates, we modeled RSV‐ARI and hospitalization incidence as attack rates. We also extracted data on authors, title, year of publication, study location, RSV season, RSV case definition, clinical specimen, diagnostic method, and participants' age group. The risk of bias for the included studies was not systematically assessed.

### 2.2 Statistical analyses

The meta‐analyses were performed based on the extracted data to establish pooled estimates of RSV‐ARI and hospitalization attack rates and hCFR, following a similar approach as Shi *et al*.^12^

For all included studies, a continuity correction of 0.5 was applied if the number of RSV‐related hospitalizations was 0. When estimating the hCFR value, we excluded studies in which no deaths were reported, as in these instances, either data on deaths were not collected or the study design (e.g., the sample size) was not appropriate to capture deaths.

A random effects model was used to assess point estimates due to between‐study data heterogeneity (DerSimonian–Laird method). The Hartung–Knapp method was used to adjust test statistics and confidence intervals (CIs).^15^ Leave‐one‐out sensitivity analyses were performed for each outcome to assess how each individual study affected the pooled estimate.

Data were analyzed in R (R Foundation for Statistical Computing, Vienna, Austria), version 3.4.3, using the meta and metafor packages.^16^, ^17^

### 2.3 Population‐based estimates of RSV disease in adults ≥60 years of age in high‐income countries

Point estimates resulting from the meta‐analyses were applied to 2019 population estimates of ≥60‐year‐old persons (the last year with reliable estimates for high‐income countries, Europe [including the five largest countries in Europe], the United States, and Japan) to estimate the number of RSV‐ARI infections, hospitalizations, and in‐hospital deaths. The 2019 population data were obtained from the UN Department of Economic and Social Affairs^18^ and from the US Census Bureau.^19^

## 3 RESULTS

### 3.1 Systematic literature review

**FIGURE 1:**
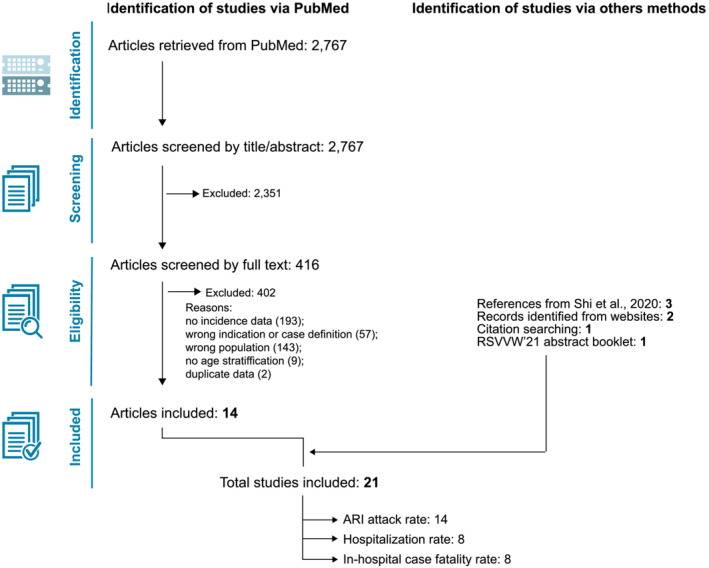
PRISMA flow diagram of the systematic literature review. ARI, acute respiratory infection; RSVVW'21, RSV Vaccine for the World 2021 conference

From 2767 records identified in the systematic literature search, we included 14 articles in the meta‐analysis,^1^, ^5^, ^20^, ^21^, ^22^, ^23^, ^24^, ^25^, ^26^, ^27^, ^28^, ^29^, ^30^, ^31^ supplemented with data from seven studies identified via citation search (Fowlkes *et al* ^32^), extraction from the Shi *et al* meta‐analysis^12^ (unpublished studies SP‐RSV11 and SP‐FIM12 and Falsey 2008–2011 data), gray literature (Novavax E201^33^ and E301 studies^34^, ^35^), and the RSVVW'21 abstract booklet (Devadiga *et al* data^4^). Of the 21 identified studies, 14 reported data on attack rates, eight on hospitalization rates, and eight on hCFR of RSV‐ARI (Figure 1).

The articles from Tseng *et al* ^36^ and Sieling *et al* ^37^ were not included as they reported the same data as in Ackerson *et al* ^20^ and Branche *et al*,^23^ respectively.

More detailed characteristics of included studies are summarized in Table S2.

### 3.2 RSV‐ARI attack rate

**FIGURE 2:**
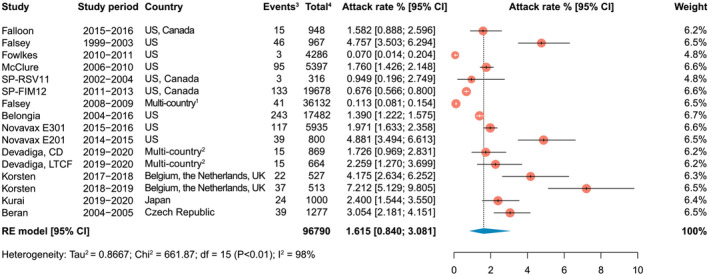
Attack rate (Attack rate was defined as the number of new cases of RSV‐associated acute respiratory infection during a specified time interval divided by the size of the population at risk. ^1^Canada, Mexico, Belgium, Czech Republic, Estonia, France, Germany, Norway, Poland, Romania, Russia, the Netherlands, the United Kingdom, and Taiwan; ^2^The United States, Belgium, Germany, Estonia, Spain, and the United Kingdom; ^3^Events, number of RSV‐associated acute respiratory infection cases; ^4^Total, total sample size of the study.) of RSV‐associated acute respiratory infections in adults aged 60 years and older. CD, community‐dwelling adults; CI, confidence interval; df, degrees of freedom; LTCF, adults living in long‐term care facilities; RE, random effects; RSV, respiratory syncytial virus; UK, United Kingdom; US, United States

To compute the pooled estimate for RSV‐ARI attack rate, 14 studies were included in the meta‐analysis, of which eight were identified through the systematic literature search^1^, ^5^, ^21^, ^25^, ^26^, ^27^, ^29^, ^30^ and six via other methods (Devadiga *et al*,^4^ Fowlkes *et al*,^32^ Novavax E201^33^ and E301,^34^, ^35^ SP‐RSV11 and SP‐FIM12^12^) (Figure 2). The prospective cohort studies by Devadiga *et al* ^4^ and Korsten *et al* ^30^ each provided data points from two separate population groups. The study by Devadiga *et al* included both community‐dwelling adults (≥60‐year‐olds) and adults living in long‐term care facilities (≥65‐year‐olds). In the study by Korsten *et al*, as part of the Respiratory Syncytial virus Consortium in Europe (RESCEU), two separate cohorts of community‐dwelling ≥60‐year‐old participants were each followed for one RSV season (2017–2018 for the first cohort and 2018–2019 for the second cohort).

The pooled attack rate of RSV‐ARI was 1.62% (95% CI: 0.84–3.08; range: 0.07–7.21) in ≥60‐year‐old adults. Leave‐one‐out sensitivity analysis showed that no single study disproportionally impacted the overall estimate (Figure S1).

### 3.3 RSV‐ARI hospitalization rate

To compute the pooled estimate for RSV‐ARI hospitalization attack rate, eight studies were included in the meta‐analysis, of which five were obtained from the systematic literature search^5^, ^23^, ^26^, ^28^, ^29^ and three (SP‐RSV11, SP‐FIM12, and Falsey 2008–2011) described in Shi *et al*.^12^ The study from Branche *et al* ^23^ provided six separate population groups from three hospitals (two in Rochester and one in New York City) during three consecutive RSV seasons.

**FIGURE 3:**
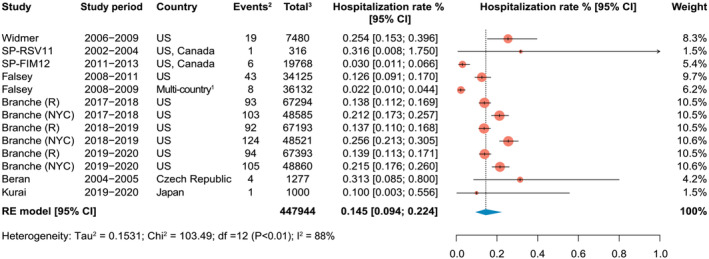
Hospitalization rate (Modeled as attack rate [defined as the number of new hospitalizations of RSV‐associated acute respiratory infection during a specified time interval divided by the size of the population at risk]. ^1^Canada, Mexico, Belgium, Czech Republic, Estonia, France, Germany, Norway, Poland, Romania, Russia, the Netherlands, the United Kingdom, and Taiwan; ^2^Events, number of hospitalizations for RSV‐associated acute respiratory infection; ^3^Total, total sample size of the study.) of RSV‐associated acute respiratory infections in adults aged 60 years and older. CI, confidence interval; df, degrees of freedom; NYC, New York City; R, Rochester (New York); RE, random effects; RSV, respiratory syncytial virus; UK, United Kingdom; US, United States.

The pooled RSV‐ARI hospitalization attack rate was estimated at 0.15% (95% CI: 0.09–0.22; range: 0.02–0.32) (Figure 3).

Leave‐one‐out sensitivity analyses showed that no single study disproportionally impacted the overall estimate (Figure S2).

### 3.4 RSV‐ARI in‐hospital case fatality rate

**FIGURE 4:**
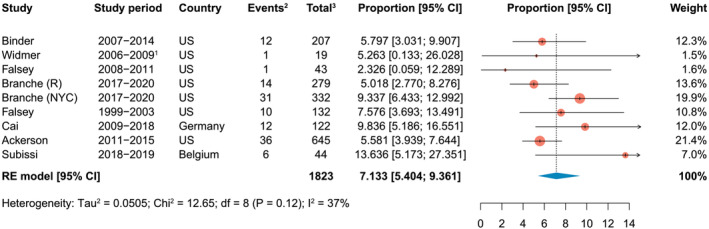
In‐hospital case fatality rate among RSV‐associated acute respiratory infections in adults aged 60 years and older. ^1^Based on the proportion of patients aged 50–64 and ≥65 years, one death was estimated in adults aged ≥60 years. This was rounded down in order to not overestimate the deaths in the ≥65 age group. ^2^Events, number of in‐hospital deaths among RSV‐associated acute respiratory infection cases; ^3^Total, number of individuals hospitalized for RSV‐associated acute respiratory infection in the study. CI, confidence interval; df, degrees of freedom; NYC, New York City; R, Rochester (New York); RE, random effects; RSV, respiratory syncytial virus; US, United States

Based on eight studies, of which seven were identified from the systematic literature search^1^, ^20^, ^22^, ^23^, ^24^, ^28^, ^31^ and one (Falsey 2008–2011) described in Shi *et al*,^12^ the hCFR in adults ≥60‐year‐olds was estimated to be 7.13% (95% CI: 5.40–9.36; range: 2.33–13.64) (Figure 4). The number of in‐hospital deaths that occurred in the study from Branche *et al* ^23^ across the three RSV seasons in patients from Rochester and New York City hospitals were obtained from unpublished data provided by the authors.

Leave‐one‐out sensitivity analysis showed that no single study disproportionally impacted the overall estimate (Figure S3).

### 3.5 Population‐based estimates of RSV disease

**FIGURE 5:**
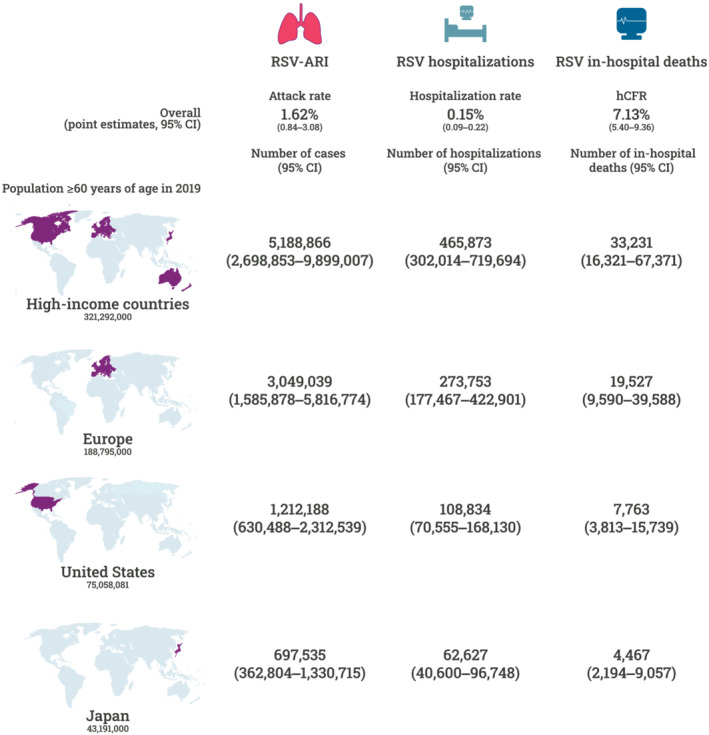
Estimated cases, hospitalizations, and in‐hospital deaths due to RSV‐associated acute respiratory infections among adults aged 60 years and older per region, 2019 population (Population data obtained from the United Nations [UN] Department of Economic and Social Affairs^18^ and the United States Census Bureau.^19^ High‐income countries were defined as “More developed regions” by the UN.). ARI, acute respiratory infection; CI, confidence interval; hCFR, in‐hospital case fatality rate; RSV, respiratory syncytial virus

We applied the above pooled estimates from the meta‐analyses to population estimates from high‐income countries, Europe, the United States, and Japan using 2019 census data^18^, ^19^ to estimate the overall burden of RSV‐ARI cases, hospitalizations, and in‐hospital deaths in that year (Figure 5). Comparable estimates for the five largest European countries (Germany, Italy, France, the United Kingdom, and Spain) are provided in Figure S4. In 2025, the number of RSV‐ARI cases, hospitalizations, and in‐hospital deaths in high‐income countries could reach 5.7 million (95% CI: 3.0 million–10.9 million), 510,000 (95% CI: 330,000–790,000), and 37,000 (95% CI: 18,000–74,000), respectively.

## 4 DISCUSSION

This systematic review summarized and estimated the burden of RSV in older adults aged ≥60 years. Compared with the review by Shi *et al*,^12^ our systematic review included different and more recent studies (studies published before 2000 were not included in our review), expanded the age range (≥60‐year‐olds vs. ≥65‐year‐olds) to reflect a broader definition of older adults^38^ and was restricted to high‐income countries.

We found a higher burden of RSV disease than previously described in the literature. The point estimate for RSV‐ARI attack rate was 16.2 per 1000 in our analysis versus 6.7 per 1000 in Shi *et al*,^12^ with overlapping 95% CI, and hCFR was 7.1% in our analysis versus 1.6% in Shi *et al*,^12^ with non‐overlapping 95% CI. The higher hCFR in our analysis could be explained by the inclusion of a few recent studies that reported a high proportion of RSV‐associated deaths^23^, ^24^, ^31^ and increases in PCR testing for RSV over time among hospitalized older adults.^39^, ^40^ A recent meta‐analysis on medically‐attended RSV in the United States suggested that the burden of disease may be even higher because PCR testing has an imperfect sensitivity for detecting RSV; without this adjustment, their findings were in line with our results.^41^

Several studies included in our meta‐analysis confirmed that the presence of comorbidities, such as chronic obstructive pulmonary disease, asthma or congestive heart failure, increases RSV attack rates and the risk of RSV‐related hospitalization in older adults.^1^, ^21^, ^23^, ^24^, ^26^ Of note, the negative effect of comorbidities on the risk of hospitalization due to RSV is present even in relatively lower age groups (50–64 years of age).^23^, ^42^

RSV hospitalization attack rates in our study (1.5 per 1000) were comparable with incidence rates in Shi *et al* ^12^ (1.0 per 1000, with overlapping 95% CI). Another recent review on the burden of RSV in adults from 1970 to 2017 reported similar RSV hospitalizations rates, 1.9–2.5 per 1000 population annually in the United States in ≥65‐year‐olds, though this meta‐analysis was not restricted to studies on confirmed cases of RSV.^43^ This previous report did include a few studies from Asia and Africa, regions with historically a paucity of data on RSV incidence, and estimated an incidence of RSV‐associated hospitalizations of 0.073–0.130 per 1000 population in these areas.^43^

Although different in scope, our data are closer to estimates from a multiple linear regression modeling study on the burden of RSV in ≥65‐year‐old adults in the United Kingdom between 1995 and 2009,^44^ which reported 19.5 general practice episodes per 1000, and a hospitalization rate of 1.6 per 1000 due to respiratory disease attributable to RSV. Of note, this study estimated overall RSV‐attributable mortality (0.09%) rather than RSV‐associated in‐hospital mortality. Another modeling study estimated the incidence rate of RSV‐attributable hospitalizations as 0.8 per 1000 in 65–74‐year‐olds and 2.6 per 1000 in ≥75‐year‐olds in the United States between 1997 and 2009.^45^

Using 2019 global population data, our results estimated significant numbers of RSV cases and deaths in older adults (Figure 5). Based on the UN Department of Economic and Social Affairs population estimates for 2025,^18^ the number of RSV cases in older adults in high‐income countries could be as high as 10.9 million, RSV hospitalizations as high as 0.8 million, and the number of deaths due to RSV as high as 74,000.

The ongoing pandemic of coronavirus disease 2019 (COVID‐19) has impacted RSV circulation through the introduction of non‐pharmaceutical interventions, causing an absence of the common seasonal peaks in the winter and, in some cases, a strong summer peak instead.^46^, ^47^ The impact of COVID‐19 on the number of RSV cases, hospitalizations and deaths could not be evaluated in the two studies included in our systematic review that reported data collected after the onset of the pandemic.^23^, ^26^ The first study was conducted in Japan and included data collected up to July 2020, but the Japanese RSV season was over when the pandemic started.^26^ In the second study conducted in the United States, the surveillance was terminated early in 2019–2020 due to the pandemic.^23^ Modeling studies suggest that RSV will return to its regular seasonal pattern in a couple of years.^48^ Moreover, in the first season or two after the COVID‐19 pandemic, RSV infections could surge to even higher numbers due to a decrease in immunity in the population.^46^, ^47^, ^48^ Consequently, the burden of RSV disease in ≥60‐year‐old adults may peak and be associated with a significant economic burden related to the higher healthcare resource utilization, both inside and outside the hospital.^49^ Having systematic testing for RSV in place will increase the number of correctly identified RSV cases, for which patient management can be optimized. Additionally, robust estimation of true RSV burden of disease is crucial for regulatory decision makers in supporting future vaccination policies, accurately predicting the impact of RSV in future seasons, and anticipating the healthcare system needs. If severe acute respiratory syndrome coronavirus 2 (SARS‐CoV‐2) becomes an endemic coronavirus and exhibits similar seasonality patterns as influenza and RSV, this would lead to overlapping seasons in temperate regions, which would put considerable additional strain on the healthcare systems during the winter season.^50^ This, in combination with the burden of disease estimates from the current analysis, highlights the need for RSV prophylaxis, not only in young children but also in older adults.

This analysis has several limitations. First, there remains a wide range of estimates of RSV burden of disease between individual studies. Differences with other meta‐analyses could be partially due to the methods applied, data used, case definitions, means of RSV detection (e.g., PCR test, seroconversion rate, or International Classification of Diseases, Tenth Revision [ICD‐10] codes), and included seasons. While, in most cases, our estimates overlap with ranges given in other studies, this may indicate that the burden of RSV disease in ≥60‐year‐olds is still underestimated. This hypothesis is even more likely when considering that serology testing, which was not performed in all studies included in the meta‐analysis, could potentially increase diagnostic yield for RSV in hospitalized patients by 50%.^51^ Second, although the RSV burden of disease increases with age, there was insufficient data available to stratify the analyses by age group. Also, age groups were not uniform across studies; some studies included data from ≥60‐year‐olds, others from ≥65‐year‐olds. Third, the data analyzed mostly originated from populations treated in urban academic settings; the demographic characteristics and prevalence of comorbidities of such populations—and as a consequence, the estimated RSV burden of disease—may not be nationally representative. Fourth, the hCFR does not represent the true number of deaths attributable to RSV as the proportion of people who die in the hospital differs across countries, particularly in the highest age groups where continuity of care in the home setting (house or nursing home) may be prioritized, impeding the ability to capture RSV cases. Fifth, we only included studies in which any deaths were reported for calculations of the hCFR pooled estimates. Sixth, the screening of the titles and abstracts retrieved by the literature search was only performed by one person. Seventh, no risk of bias assessment was performed. Lastly, while our meta‐analysis included studies conducted in a variety of high‐income countries, it is hard to generalize across regions because of a paucity of data.

There are some general limitations to RSV research and surveillance in older adults worldwide, which could be improved: Testing in older adults is not performed consistently, RSV is usually not a notifiable infection in older adults, there is a lack of surveillance systems with a case definition dedicated to RSV, and data from low‐ and middle‐income regions are lacking. Data from these countries would be needed to estimate the global burden of RSV disease in ≥60‐year‐old adults.

In conclusion, there is a significant burden of disease of RSV among ≥60‐year‐old adults in high‐income countries, and estimates point towards higher rates than previously reported. This likely indicates that a larger pressure will be placed on the healthcare systems as the population ages, especially in future seasons when RSV may co‐circulate with influenza and possibly SARS‐CoV‐2. Data on the RSV burden of disease in older adults are useful for guiding future prevention programs.

**FIGURE 6:**
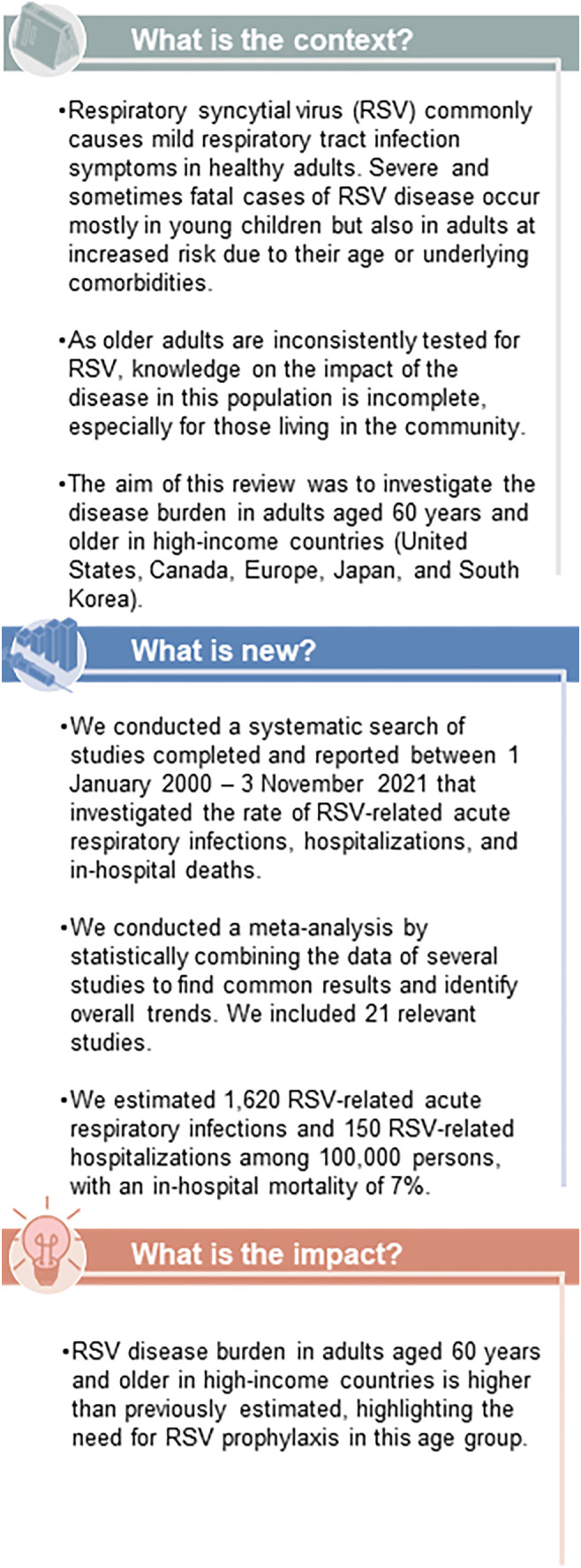
Plain language summary. RSV, respiratory syncytial virus

We provide a brief summary of our findings and the corresponding implications for non‐expert audiences in a plain language summary (Figure 6).

## CONFLICT OF INTEREST

M Savic, Y Penders and JY Pirçon are employees of the GSK group of companies. M Savic and JY Pirçon hold shares in the GSK group of companies as part of their employee remuneration. A Branche was a paid consultant for the GSK group of companies during the conduct of the work reported in the manuscript and has received grants from Merck, Janssen, Cyanvac and Pfizer outside the submitted work. All authors declare no other financial or non‐financial relationships and activities.

## AUTHOR CONTRIBUTIONS

**Miloje Savic:** Conceptualization; data curation; formal analysis; investigation; methodology; supervision. **Yolanda Penders:** Conceptualization; data curation; formal analysis; investigation; methodology; supervision. **Ting Shi:** Methodology; supervision. **Angela Branche:** Supervision. **Jean‐Yves Pirçon:** Conceptualization; data curation; formal analysis; investigation; methodology; supervision.

### PEER REVIEW

The peer review history for this article is available at https://publons.com/publon/10.1111/irv.13031.

## Supporting information


**Table S1.** Search strategy of the systematic literature search
**Table S2.** Characteristics of the included studies
**Figure S1.** Leave‐one‐out sensitivity analysis of the attack rate of RSV‐associated acute respiratory infections in adults aged 60 years and older
**Figure S2.** Leave‐one‐out sensitivity analysis of the hospitalization rate^a^ of RSV‐associated acute respiratory infections in adults aged 60 years and older
**Figure S3.** Leave‐one‐out sensitivity analysis of the in‐hospital case fatality rate among RSV‐associated acute respiratory infections in adults aged 60 years and older
**Figure S4.** Estimated cases, hospitalizations, and in‐hospital deaths due to RSV‐associated acute respiratory infections among adults aged 60 years and older per region, 2019 population

## Data Availability

The data used for this analysis are included in published materials and are thus available in the cited references. Data generated by the meta‐analysis are available from the corresponding author upon request.
