# Supplementary material for: Respiratory syncytial virus disease burden in adults aged 60 years and older in high‐income countries: A systematic literature review and meta‐analysis
Source: Influenza Other Respir Viruses. 2022 Nov 11;17(1):e13031. doi: 10.1111/irv.13031 (PMC9835463; doi:10.1111/irv.13031)
Supplement: Supplementary file 1 — Table S1. Search strategy of the systematic literature search Table S2. Characteristics of the included studies Figure S1. Leave‐one‐out sensitivity analysis of the attack rate of RSV‐associated acute respiratory infections in adults aged 60 years and older Figure S2. Leave‐one‐out sensitivity analysis of the hospitalization ratea of RSV‐associated acute respiratory infections in adults aged 60 years and older Figure S3. Leave‐one‐out sensitivity analysis of the in‐hospital case fatality rate among RSV‐associated acute respiratory infections in adults aged 60 years and older Figure S4. Estimated cases, hospitalizations, and in‐hospital deaths due to RSV‐associated acute respiratory infections among adults aged 60 years and older per region, 2019 population [file IRV-17-e13031-s001.docx]

# Supporting information

# Supplementary table 1. Search strategy of the systematic literature search

| Date of search: 01-01-2000 to 03-11-2021 |  |
| --- | --- |
| (((““Respiratory syncytial virus” OR “Respiratory syncytial virus infection” OR RSV) AND (“aged” OR “elderly” OR older adulthood OR older adults OR older adults care OR older adult OR “adult”)) AND ((“Incidence” OR inciden* OR “Prevalence” OR prevalen* OR frequency OR frequencies OR rate* OR proportion* OR distribut*) OR (“Morbidity” OR morbidity OR complication* OR “Mortality” OR mortality OR mortalities OR “Death” OR death* OR case-fatalit* OR lethal* OR died OR severe*)) AND (“2000/01/01”[Date – Publication] : “2021/11/03”[Date – Publication])) | 2767 |

# Supplementary table 2. Characteristics of the included studies

| **Reference** | **Location** | **Seasons** | **Case definition** | **Clinical specimen** | **Diagnostic tests** | **Age group (years)** | **Attack rate** | **Hospit. rate** | **hCFR** |
| --- | --- | --- | --- | --- | --- | --- | --- | --- | --- |
| Ackerson, 2019 [^20^](#_ENREF_20) | US | 2011–2015 | RSV-positive cases | N/S | PCR (92%) or culture (8%) | ≥60 |  |  | X |
| Belongia, 2018 [^21^](#_ENREF_21) | US | 2004–2016 | ARI + cough^a^ | Midturbinate swab | PCR | ≥60 | X |  |  |
| Beran, 2021 [^29^](#_ENREF_29) | Czech Republic | 2004–2005^b^ | ARI | Nasal swab or blood | PCR or serology | ≥65 | X | X |  |
| Binder, 2017 [^22^](#_ENREF_22) | US | 2007–2014 | RSV-positive cases | Nasopharyngeal swab | Dipstick immunoassay | ≥60 |  |  | X |
| Branche, 2021 [^23^](#_ENREF_23) | US | 2017–2020 | ARI | Nasopharyngeal swab or midturbinate swab | PCR | ≥65 |  | X | X^c^ |
| Cai, 2020^d^ [^24^](#_ENREF_24) | Germany | 2009–2018 | SARI | N/S | N/S | ≥65 |  |  | X |
| Devadiga, 2021 [^4^](#_ENREF_4) | US, Belgium, Germany, Estonia, Spain, UK | 2019–2020 | ARI | Nasal and throat swab or blood | PCR or ELISA | ≥60 (≥65 for LTCF residents) | X |  |  |
| Falloon, 2017 [^25^](#_ENREF_25) | US/Canada^e^ | 2015–2016 | ARI | Mid-turbinate swab, sputum | PCR | ≥60 | X |  |  |
| Falsey, 2005 [^1^](#_ENREF_1) | US | 1999–2003 | ARI | Nasopharyngeal swab or blood | PCR, culture, or serology | ≥65^f^ | X |  | X |
| Falsey, 2014 [^5^](#_ENREF_5) | Canada, Mexico, US^g^, Belgium, Czech Republic, Estonia, France, Germany, Norway, Poland, Romania, Russia, the Netherlands, UK, and Taiwan | 2008–2009 | ILI | Nasal and throat swab | PCR | ≥65 | X | X |  |
| Falsey^h^ | US | 2008–2011 | ARI | Nasal swab, sputum | PCR | ≥65 |  | X | X |
| Fowlkes, 2014 [^32^](#_ENREF_32) | US | 2010–2011 | ILI or ARI | Nasal, nasopharyngeal or oropharyngeal swab | PCR | ≥65 | X |  |  |
| Korsten, 2021 [^30^](#_ENREF_30) | Belgium, the Netherlands, UK | 2017–2019 | ARI | Nasopharyngeal swab or blood | PCR or serology | ≥60 | X |  |  |
| Kurai, 2021 [^26^](#_ENREF_26) | Japan | 2019–2020 | ARI | Nasopharyngeal swab | PCR | ≥65 | X | X |  |
| McClure, 2014 [^27^](#_ENREF_27) | US | 2006–2010 | Fever, chills, or cough | Nasopharyngeal swab | PCR | ≥60 | X |  |  |
| Novavax E201 [^33^](#_ENREF_33) | US | 2014–2015 | ARI | N/S | PCR | ≥60 | X |  |  |
| Novavax E301 [^34^](#_ENREF_34)^,^[^35^](#_ENREF_35) | US | 2015–2016 | ARI | N/S | PCR | ≥60 | X |  |  |
| Subissi, 2020 [^31^](#_ENREF_31) | Belgium | 2018–2019 | SARI | Nasopharyngeal swab or aspirate | PCR | ≥65 |  |  | X |
| SP-RSV11^h^ | US/Canada | 2002–2004 | ARI | Nasopharyngeal swab or blood | PCR | ≥65 | X | X |  |
| SP-FIM12^h^ | US/Canada | 2011–2013 | ARI | Nasopharyngeal swab or blood | PCR | ≥65 | X | X |  |
| Widmer, 2012 [^28^](#_ENREF_28) | US | 2006–2009 | ILI or ARI | Nasal and throat swab | PCR | ≥65 |  | X | X |

^a^Case definition varied by season but included fever/feverishness or cough during most seasons; ^b^The 2003–2004 season was also assessed in this study but this was excluded from our analysis because no RSV cases were reported; ^c^The number of in-hospital deaths that occurred across the three RSV seasons in patients from Rochester and New York City hospitals were obtained from unpublished data provided by the authors; ^d^Based on hospital surveillance data utilizing the ICD-10 (International Statistical Classification of Diseases and Related Health Problems 10^th^ Revision) codes to identify RSV-positive patients; ^e^US and Canada mentioned in the inclusion criteria. Primarily in the US but also in Canada, Eastern Europe, Chile, and South Africa. The percentage of participants out of scope is not known; ^f^Includes healthy patients (mean age ± standard deviation [SD]: 75 ± 6 years) and high-risk patients (mean age ± SD: 70 ± 11 years); ^g^Samples from US subjects were not included in the present study as ethics approval for additional virologic studies would have been required; ^h^Unpublished study reported in Shi et al. [^12^](#_ENREF_12). These studies are referred to as US, NY, Monroe (Falsey) US & Canada, 40 sites (SP-RSV11), US & Canada, 126 sites (SP-FIM12) in Shi et al.[^12^](#_ENREF_12)

ARI, acute respiratory infection; ELISA, enzyme-linked immunosorbent immunoassay; hCFR, in-hospital case fatality rate; Hospit., hospitalization; ILI, influenza-like illness; LTCF, long-term care facility; N/S, not specified; PCR, polymerase chain reaction; RSV, respiratory syncytial virus; SARI, severe acute respiratory infection; UK, United Kingdom; US, United States.

# Supplementary figure 1. Leave-one-out sensitivity analysis of the attack rate of RSV-associated acute respiratory infections in adults aged 60 years and older


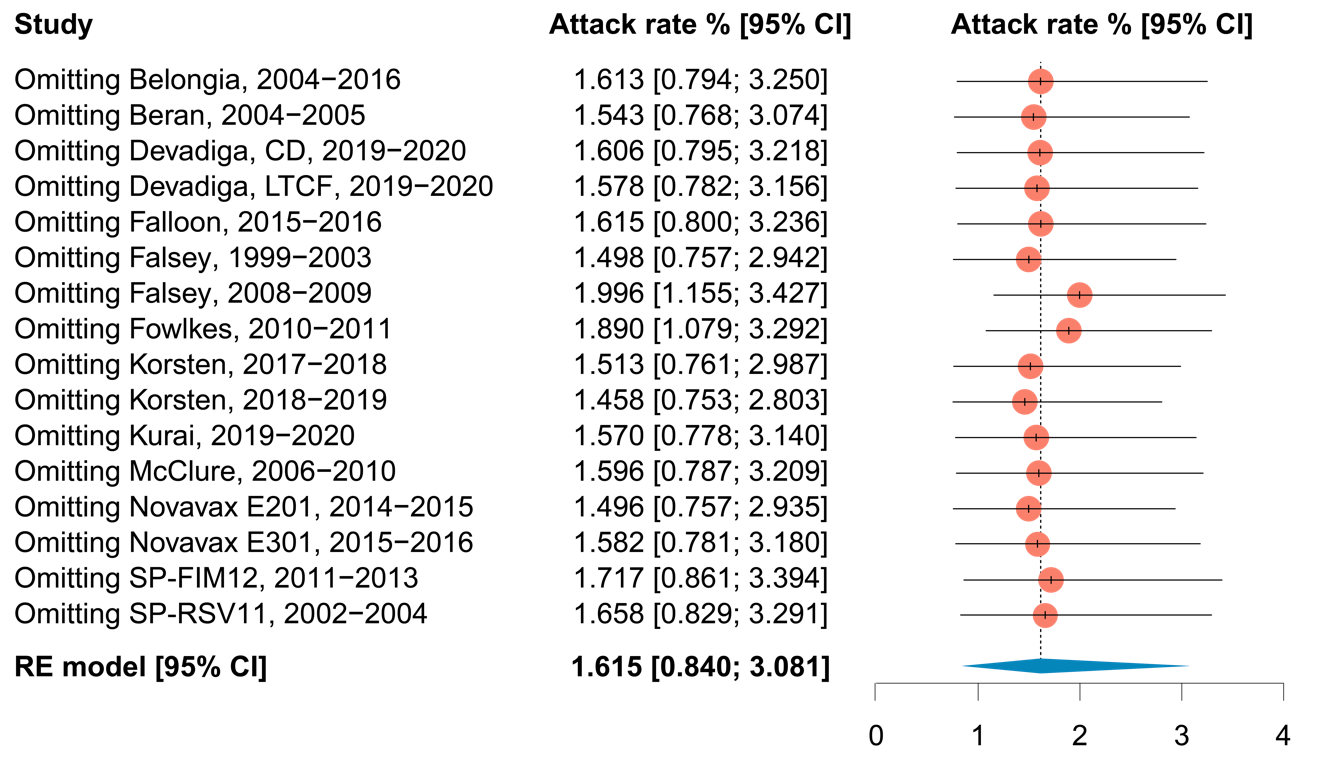


CD, community-dwelling adults; CI, confidence interval; LTCF, adults living in long-term care facilities; RE, random effects; RSV, respiratory syncytial virus.

# Supplementary figure 2. Leave-one-out sensitivity analysis of the hospitalization rate^a^ of RSV-associated acute respiratory infections in adults aged 60 years and older


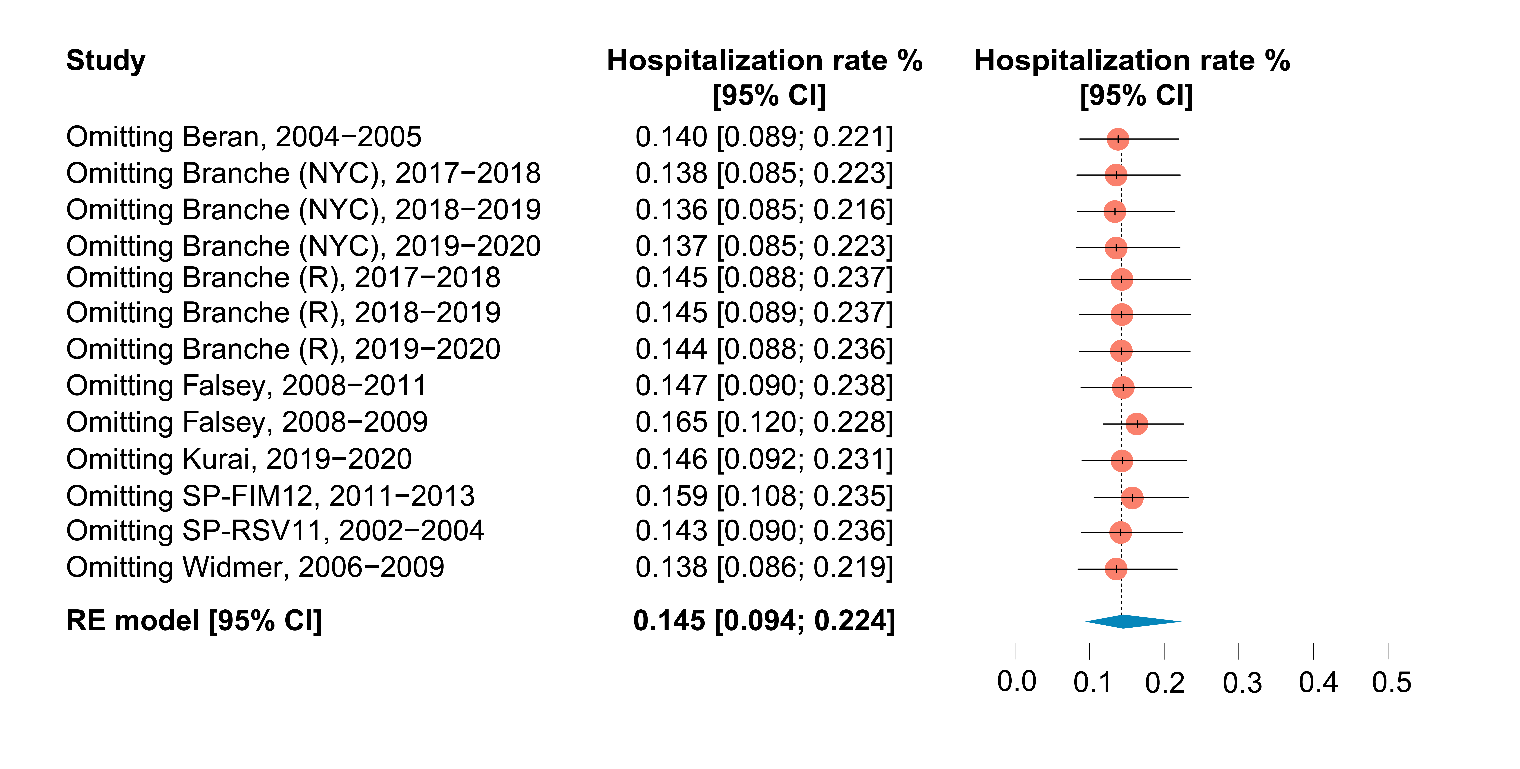


^a^Modeled as attack rate (defined as the number of new hospitalizations of RSV-associated acute respiratory infection during a specified time interval divided by the size of the population at risk). CI, confidence interval; NYC, New York City; R, Rochester (New York); RE, random effects; RSV, respiratory syncytial virus.

# Supplementary figure 3. Leave-one-out sensitivity analysis of the in-hospital case fatality rate among RSV-associated acute respiratory infections in adults aged 60 years and older


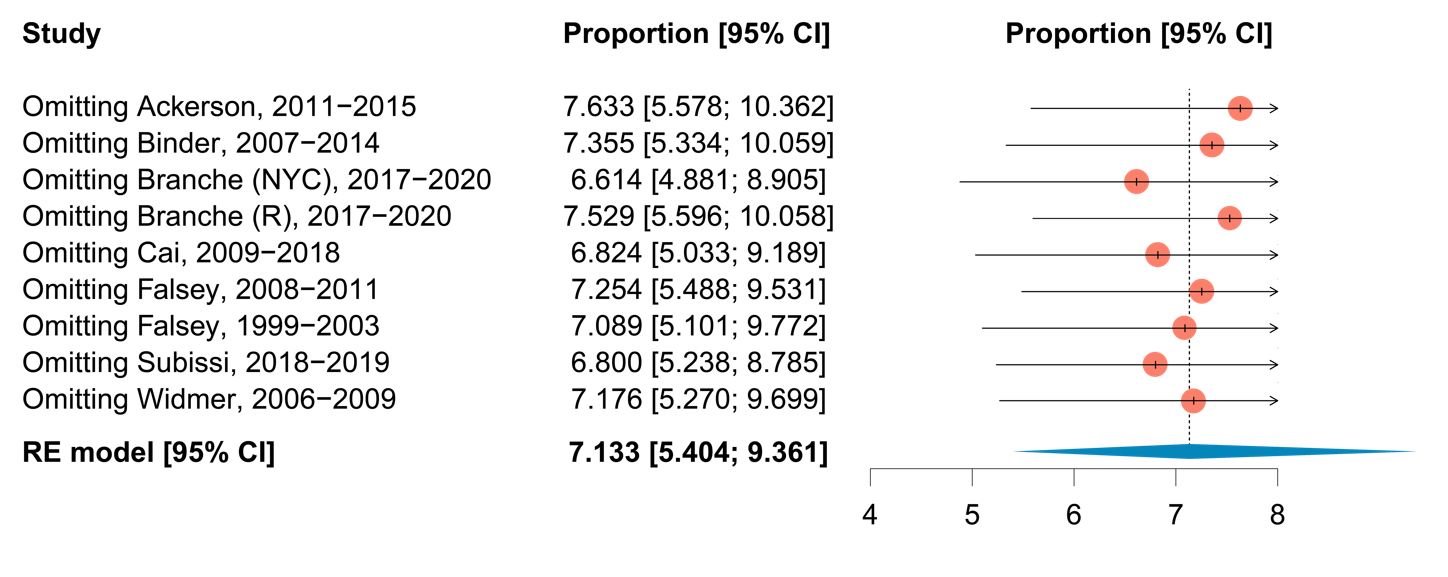


CI, confidence interval; NYC, New York City; R, Rochester (New York); RE, random effects; RSV, respiratory syncytial virus.

# Supplementary figure 4. Estimated cases, hospitalizations, and in-hospital deaths due to RSV-associated acute respiratory infections among adults aged 60 years and older per region, 2019 population^a^


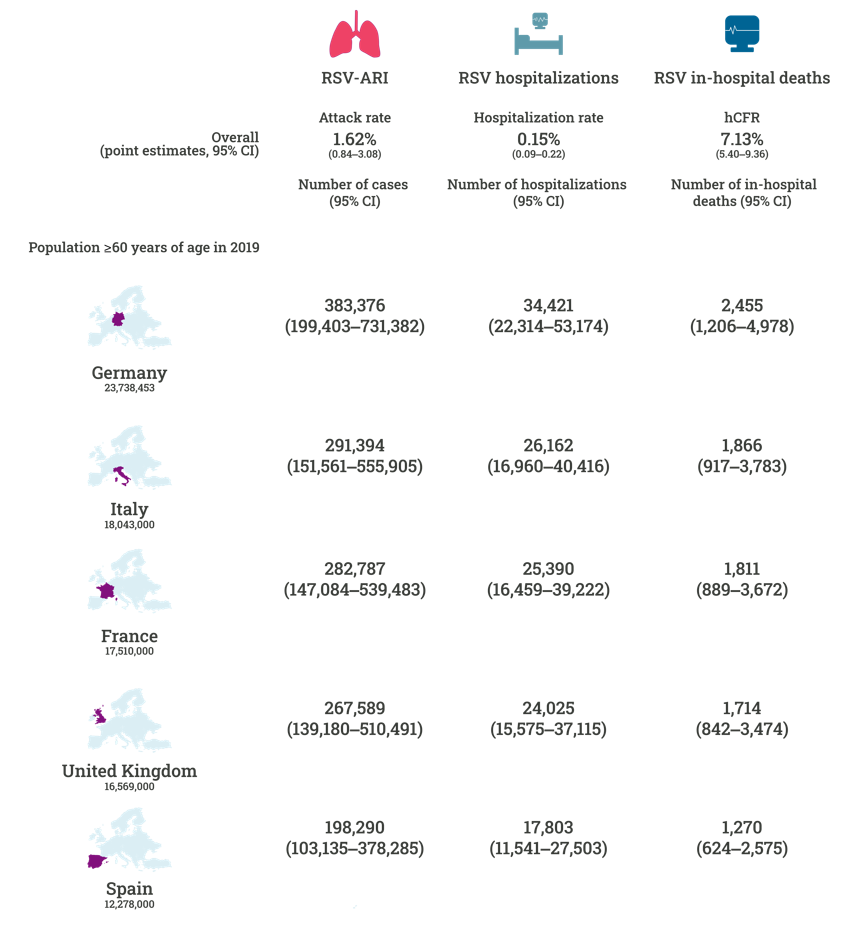


^a^Population data obtained from the United Nations Department of Economic and Social Affairs [^18^](#_ENREF_18).

ARI, acute respiratory infection; CI, confidence interval; hCFR, in-hospital case fatality rate; RSV, respiratory syncytial virus.
